# Supplementary material for: ARPIR: automatic RNA-Seq pipelines with interactive report
Source: BMC Bioinformatics. 2020 Dec 21;21(Suppl 19):574. doi: 10.1186/s12859-020-03846-2 (PMC7751108; doi:10.1186/s12859-020-03846-2)
Supplement: Supplementary file 1 — Additional file 1. Detailed description of the steps addressed during the analysis. [file 12859_2020_3846_MOESM1_ESM.docx]

**SUPPLEMENTARY MATERIAL**

**ARPIR: Automatic RNA-Seq Pipelines with Interactive Report**

Giulio Spinozzi^1*^, Valentina Tini^1^, Alessia Adorni^1^, Brunangelo Falini^1^ and Maria Paola Martelli^1*^

^1^University of Perugia, Department of Medicine, Section of Hematology

*Email of Corresponding author: [giulio.spinozzi@unipg.it](mailto:giulio.spinozzi@unipg.it), [maria.martelli@unipg.it](mailto:maria.martelli@unipg.it)

**TABLE OF CONTENTS**

[**1. COMPARISONS BETWEEN DIFFERENT TOOLS** 3](#_Toc25834232)

[**2. COMPARISONS BETWEEN DIFFERENT PIPELINES** 5](#_Toc25834233)

[**3. SIMULATIONS** 8](#_Toc25834234)

[**4. AUTOMATIC RNA-SEQ PIPELINES WITH INTERACTIVE REPORT (ARPIR)** 10](#_Toc25834235)

[**4.1 FastQ quality** 10](#_Toc25834236)

[**4.2 Pre-processing** 11](#_Toc25834237)

[**4.3 Alignment** 12](#_Toc25834238)

[**4.4 BAM quality** 13](#_Toc25834239)

[**4.5 Quantification** 14](#_Toc25834240)

[**4.6 Differential Expression Analysis** 14](#_Toc25834241)

[**4.7 Gene Ontology analysis** 15](#_Toc25834242)

[**4.8 Pathway analysis** 15](#_Toc25834243)

[**4.9 Shiny report** 15](#_Toc25834244)

[**REFERENCES** 17](#_Toc25834245)

# **1. COMPARISONS BETWEEN DIFFERENT TOOLS**

We have considered several tools for RNA-Seq data analysis to evaluate the options offered by each one [1]. In addition to quality control on the files produced, another important aspect is represented by the final tertiary-analysis, aimed at searching for a biological meaning in the results obtained. To make the tool easy to use, the presence of a graphical interface, as well as the possibility of displaying the results in an interactive report, is helpful. Finally, also the possibility of exploiting different pipelines can be useful to compare the results or to allow to choose specific tools. These were therefore the parameters we considered. Of the 29 pipelines reported, however, only 10 were useful for this comparison, while most were excluded due to broken links or because they were not specific for a complete RNA-Seq data analysis (Table S1). As shown in the table, although all the features examined are present in at least one tool, none of the tools presents them all apart from ARPIR.

|  | Quality control | GO | Pathway | Multiple Pipelines | GUI | Interactive Report |
| --- | --- | --- | --- | --- | --- | --- |
| ArrayExpressHTS [2] | **+** |  |  |  |  | **+** |
| BioJupies [3] |  | **+** | **+** |  | **+*** | **+** |
| BioWardrobe [4] | **+** |  |  |  | **+** | **+** |
| Chipster [5] | **+** |  |  | **+** | **+** |  |
| DEWE [6] | **+** |  |  | **+** | **+** |  |
| GENE-Counter [7] |  | **+** |  | **+** |  |  |
| GT-FAR [8] | **+** |  |  |  | **+**** |  |
| RobiNA [9] | **+** |  |  |  | **+** | **+** |
| RseqFlow [10] | **+** |  |  |  |  |  |
| TRAPLINE [11] | **+** |  |  |  | **+***** |  |
| ARPIR | **+** | **+** | **+** | **+** | **+** | **+** |
| BioQueue | It is a queue system to accelerate the proceeding of bioinformatic workflows. | | | | | |
| easyRNASeq | It calculates the coverage of high-throughput short-reads against a genome of reference. | | | | | |
| ExpressionPlot | Link does not work (Project Expired?). | | | | | |
| FASTGenomics | It works on single-cell RNA sequencing data. | | | | | |
| FX | Link does not work (Project Expired?). | | | | | |
| Galaxy | It is a framework, not an RNA-Seq specific tool. | | | | | |
| GenePattern | Link does not work (Project Expired?). | | | | | |
| GeneProf | Server down for maintenance. | | | | | |
| GREIN | It works only on GEO data. | | | | | |
| MultiExperiment Viewer | Link does not work (Project Expired?). | | | | | |
| NGSUtils | Link does not work (Project Expired?). | | | | | |
| Rail-RNA | Scalable analysis of RNA-Seq splicing and coverage. | | | | | |
| RAP | Absence of a link. | | | | | |
| RSEQtools | It is not a pipeline but a whole of modules. | | | | | |
| S-MART | It handles mapped RNA-Seq data. | | | | | |
| Taverna | It is not an RNA-Seq specific tool. | | | | | |
| TCW | It is not a pipeline but a whole of different modules. | | | | | |
| ViennaNGS | A Perl extension and collection of utilities. | | | | | |
| wapRNA | Link does not work (Project Expired?). | | | | | |

Table S1: Comparison table of different tools for RNA-Seq analysis. Only tools that included the use of a pipeline for RNA-Seq data analysis were taken into consideration. In other cases, the reason for the exclusion is reported. * Allows to upload data up to 5 GB, **Amazon based cloud solution, *** Works on Galaxy

# **2. COMPARISONS BETWEEN DIFFERENT PIPELINES**

To evaluate the different performance of the eight pipelines, we analyzed six samples of the IMS-M2 cell line [12]. It is a cell line of Acute Myeloid Leukemia (AML) characterized by the mutation of the *NPM1* gene. The samples belonged to two groups, one treated and one untreated, used as a control. Each of the two groups presented three replicates. The kit used for the preparation of the sample was the TruSeq RNA [13], which leads, starting from an RNA sample, to the formation of a library compatible with Illumina sequencers. The sequencer used for sequencing was HiSeq 2500 by Illumina [14]. It provides two speed modes for sequencing, rapid run and high-output run, and can also be configured to work with a flow cell alone or with two at the same time. In our case the experiment was conducted in rapid run and with a flow cell. Sequencing occurred in paired-end and using two lanes per sample. The two lanes corresponding to the same sample have been merged into a single file before the alignment phase.

The computer used to perform the analysis and to evaluate the times and peaks of RAM was a HP Z840 Workstation with Intel(R) Xeon(R) CPU E5-2690 v4 @ 2.60GHz processor and 256 Gb 2400 MHz DDR4 RAM memory. The processes were carried out with the use of 4 threads.

The time taken was calculated for a single sample in the phases in which it was possible, and then multiplied by the six samples in question.

In the *TopHat2-Cufflinks-cummeRbund* [15-17] pipeline the alignment phase took about 5 hours per sample and reached a RAM peak of 3.3 Gb during the alignment phase on the genome. The quantification phase involved the consecutive use of *cufflinks*, *cuffmerge* and *cuffquant*, took about 40 minutes per sample and reached a peak of 800 Mb during the expression level quantification of *cuffquant*. Finally, the differential analysis was performed with *cuffdiff* [18] and the results shown with *cummeRbund*. Overall it took about 40 minutes and reached a peak of 2.9 Gb during the testing for differential expression and regulation of *cuffdiff*. Overall, the pipeline took about 35 hours to analyze the six samples and the highest RAM peak was 3.3 Gb achieved during the alignment.

In the *HISAT2-StringTie-Ballgown* [19-21] pipeline the alignment phase is followed by the conversion of the SAM file into BAM, the sorting and the indexing, all done through *samtools* [22]. Overall this phase took about 35 minutes per sample and reached the highest peak with 4.3 Gb during alignment with *HISAT2*. Quantification with *StringTie* took about 4 minutes per sample and reached a peak of 200 Mb. Finally, the DEA with *Ballgown* took 1 minute and reached 1.2 Gb of RAM. As a whole, the pipeline took about 4 hours to analyze the six samples and reached the highest peak at 4.3 Gb with *HISAT2*.

The *HISAT2-featureCounts-DESeq2* [23, 24] pipeline took the same time and reached the same RAM memory peaks as the previous one in the alignment phase, while it took about 24 minutes for all six samples in the quantification phase with *featureCounts* and reached a peak of 350 Mb of RAM. Instead, the differential analysis with *DESeq2* required only few seconds and reached a peak of 1.2 Gb. Overall, the pipeline took about 4 hours to analyze six samples and reached the highest RAM peak with 4.3 Gb during alignment.

The *HISAT2-featureCounts-edgeR* [25, 26] pipeline largely coincides with the previous one and differs only in the differential analysis with *edgeR*, which again required few seconds and reached a peak of 1.3 Gb of RAM. Overall, the pipeline took about 4 hours for six samples and reached the peak of RAM at 4.3 Gb during alignment.

The STAR pipelines (*STAR-Cufflinks-cummeRbund* [16, 17, 27], *STAR-featureCounts-DESeq2* [23, 24, 27], *STAR-featureCounts-edgeR* [23, 25, 27]) are characterized by a time consumption slightly lower than that of the *HISAT2* pipelines, but require reaching 30Gb RAM peaks in order to work at their best. In the alignment part they take about three and a half hours for six samples, while in the quantification and differential analysis part they coincide with the previous ones.

The *kallisto-sleuth* [28, 29] pipeline took about 20 minutes per sample for the pseudo-alignment and quantification phase and reached a RAM peak of 1.4 Gb. The differential analysis phase with *sleuth* took just over 2 minutes and reached a peak of 2.2 Gb of RAM. Overall, the pipeline took about 2 hours to analyze the six samples and reached its peak at 2.2 Gb of RAM in the differential analysis.

The plot below (Figure S1) shows the results of time and RAM peaks for the eight pipelines.


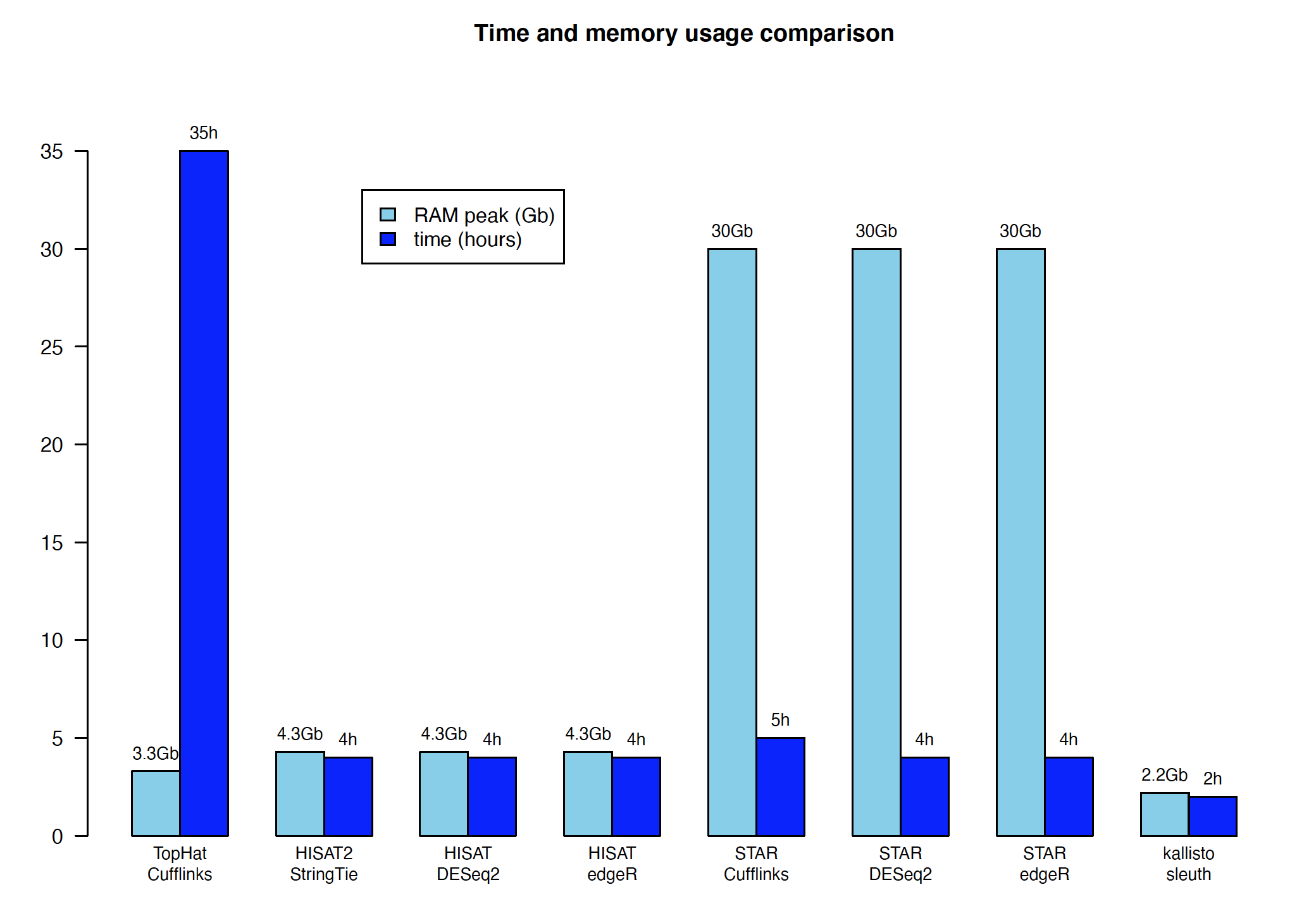


Figure S1. Histogram of the times and the RAM memory peaks reached during the RNA-Seq analysis for the eight pipelines. RAM peaks are similar for all pipelines and less than 5Gb, whit the exception of STAR pipelines, which reach a peak of 30Gb during the alignment step. Regarding the time, however, that of TopHat2 is the longest pipeline with 35 hours for six samples, while the others remain under 4 hours.

By first evaluating the pipelines in terms of time consumption and memory usage peaks achieved, it was found that the most expensive step is in all cases the alignment, except in the *kallisto* pipeline, where higher RAM peaks are reached in the differential analysis. So, comparing the four different aligners that we have used, it is clear that the slowest is *TopHat2*, which requires more than five hours per sample for alignment only. With *HISAT2* it comes down to about 15 minutes per sample, followed by the longest conversion steps of the SAM file in BAM, sorting and indexing, which increase the time for a sample up to 40 minutes. *STAR* requires similar times, although slightly less than those of *HISAT2*. *Kallisto*, on the other hand, takes about 20 minutes per sample for pseudo-alignment and quantification. As for the consumption of memory, the highest are with *STAR*, with 30Gb, then down to 4.3Gb and 3.3Gb with *HISAT2* and *TopHat2* respectively up to 1.4Gb with *kallisto*, which, using an alignment on the transcriptome, requires consumption of memory lower than the alignments made on the genome. As for the quantification methods, instead, *featureCounts* with its read count requires slightly shorter times than the quantification with the statistical approach of *Cufflinks* and *StringTie*. The consumption of memory, however, never exceeds few hundred megabytes. The differential analysis in *R*, finally, required in all cases only few minutes or few seconds and did not have much influence in the overall time.

From the point of view of memory consumption, all pipelines are quite similar, with the highest peaks concentrating in the initial alignment phase. The only exception is STAR, which requires 30Gb of RAM in order to function at its best.

We then considered the results and for a first summary comparison we evaluated the differentially expressed genes common to the eight pipelines. Genes were considered differentially expressed with a *p*-value adjusted lower than 0.05 and an absolute value of log2-Fold Change higher than 1.5. The results for the eight pipelines are shown in the following table (Table S2).

|  | **Genes unique for the pipeline** | **Total** |
| --- | --- | --- |
| **HISAT2-StringTie-Ballgown** | 207 | 440 |
| **HISAT2-featureCounts-DESeq2** | 16 | 898 |
| **STAR-featureCounts-DESeq2** | 16 | 886 |
| **HISAT2-featureCounts-edgeR** | 7 | 645 |
| **STAR-featureCounts-edgeR** | 3 | 645 |
| **TopHat2-Cufflinks-cummeRbund** | 25 | 523 |
| **STAR-Cufflinks-cummeRbund** | 33 | 493 |
| **Kallisto-sleuth** | 1817 | 2195 |

Table S2. The table shows for each of the eight pipelines the total number of differentially expressed genes identified and the number of genes identified exclusively in that pipeline and not in others. Kallisto has the highest number, but most of the genes are not confirmed by the other pipelines. Those with edgeR are the two pipelines with the lowest number of these potential false positives.

Regarding the results of the differential expression compared between the eight pipelines, looking only at the number of genes that are significantly expressed compared with the control, *kallisto* provides the largest number, with over 2,000 genes, followed by *DESeq2* with 898 and 886 genes. Looking at the results of *kallisto*, however, it emerges that more than 80% of the identified genes are unique to the pseudo-alignment method and are not supported by the other methods. This suggests that they are therefore false positives. The pipeline that has instead identified the least number of genes differentially expressed is that of *HISAT2-StringTie-Ballgown,* with less than 500 genes. Even in this case, however, half of the identified genes is unique to this method and therefore allows us to suppose that also these are false positives. Better results are obtained with *DESeq2*, which has about 500 significant genes also confirmed by other methods, but another 200 are not in common (most of these genes are instead shared in the two pipelines that use *DESeq2*, down to 16 the number of genes not in common). *edgeR* gives results similar to *DESeq2*, but the number of significant genes is reduced to 500 and almost all of them are in common with the other methods (also excluding one of the two pipelines that shares the differential analysis method). Finally, even with the *Cufflinks-cummeRbund* pipelines good results are obtained from the differential analysis, with about 500 genes, most of which in common with the other methods.

These are obviously inaccurate results, as we do not know in advance the genes that are actually expressed differentially between the two groups. Precisely for this reason, the next step was to repeat the analysis using simulated RNA-Seq data, as described below.

# **3. SIMULATIONS**

To validate the results obtained from the eight pipelines, we have resorted to simulation of RNA-Seq reads. The use of simulated data allows the a priori knowledge of the differentially expressed genes between the two groups and therefore allows to calculate sensitivity and specificity of the eight pipelines taken into consideration. The simulated reads were obtained through the R package *polyester* [30]. We started from chromosome 22 only (hg19) and we selected 918 transcripts for a total of 547 genes. The simulated reads are 100 in length and paired-end. They were also generated considering a coverage of ~20x calculated as

$reads for transcript= 20*\frac{transcript length}{read length}$.

The package *polyester* assigns a baseline level of expression for each transcript that is then multiplied by the chosen Fold Change values, in our case random values between 1 and 4. Two categories have been considered, one of hypothetical treated and one of hypothetical control, each consisting of three replicates. The number of genes that is differentially expressed is equal to 285 out of the total of 547.

The code used to generate the simulated reads is as follows:

library(polyester)

library(Biostrings)

fasta_file = system.file('extdata', 'chr22.fa', package='polyester')

fasta = readDNAStringSet(fasta_file)

readspertx = round(20 * width(fasta) / 100)

fold_changes = matrix(sample(c(1,1,1,1,1,1,1,1,1,2,3,4),918*2,replace=T), nrow=918)

simulate_experiment('chr22.fa', reads_per_transcript=readspertx, gzip=T,

num_reps=c(3,3), fold_changes=fold_changes, outdir='simulated_reads')

Starting from the same twelve FastQ files thus obtained, the eight pipelines were carried out in parallel.

To evaluate the results obtained, we calculated sensitivity and specificity for each pipeline.

True positive were considered:

- genes that were differentially expressed both in the pipeline (*p*-value adjusted <0.05) and in the original data (difference greater than 0 between the two groups in the Fold Change multiplication matrix).

True negatives were considered:

- genes that were not differentially expressed both in the pipeline (*p*-value adjusted> 0.05) and in the original data (difference of 0 between the two groups in the Fold Change multiplication matrix).

False negatives were considered:

- genes that were not differentially expressed in the pipeline (*p*-value adjusted> 0.05) despite being differentially expressed in the original data (difference greater than 0 between the two groups in the Fold Change multiplication matrix),
- genes present in the original data (independently of the differential expression), but which did not appear at all as expressed in the pipeline data.

False positives were considered:

- genes that were differentially expressed in the pipeline (*p*-value adjusted <0.05) although not differentially expressed in the original data (difference of 0 between the two groups in the Fold Change multiplication matrix),
- genes not present in the original data, but which appear as expressed in the pipeline data (independently of the differential expression).

The sensitivity was calculated as

$sensitivity=\frac{true positive}{true positive+false negative}$

while the specificity has been calculated as

$specificity=\frac{true negative}{true negative+false positive}$.

The following table (Table S3) shows the results obtained.

|  | True positive | True negative | False positive | False negative | Sensitivity | Specificity |
| --- | --- | --- | --- | --- | --- | --- |
| HISAT_edgeR | 260 | 227 | 42 | 47 | 0.8469055 | 0.8438662 |
| STAR_ edgeR | 259 | 226 | 34 | 49 | 0.8409091 | 0.8692308 |
| HISAT_DESeq2 | 260 | 228 | 44 | 47 | 0.8469055 | 0.8382353 |
| STAR_ DESeq2 | 259 | 228 | 37 | 48 | 0.8436482 | 0.8603774 |
| HISAT_Ballgown | 233 | 225 | 64 | 64 | 0.7845118 | 0.7785467 |
| TopHat_cummeRbund | 241 | 233 | 28 | 62 | 0.7953795 | 0.8927203 |
| STAR_ cummeRbund | 241 | 227 | 37 | 62 | 0.7953795 | 0.8598485 |
| Sleuth | 235 | 196 | 105 | 96 | 0.7099698 | 0.6511628 |

Table S3. Results of sensitivity and specificity obtained from the eight tested pipelines. edgeR and DESeq2 have the lowest false negative numbers and in fact have the highest sensitivity values, while TopHat is the pipeline with the lowest false positive number and therefore has the highest specificity value.

As you can see from the table, the best pipelines are the first four, those of *STAR* and *HISAT2* with *featureCounts* for quantification. The pipelines that use *Cufflinks* for quantification and *cummeRbund* for DEA, instead show better results in specificity, but worse in sensitivity. *Ballgown* and *sleuth*, on the other hand, show mediocre results both in terms of sensitivity and specificity. In particular for *Ballgown*, the responsibility for such performance can be attributed to quantification and differential analysis, as it shares the alignment with the other *HISAT2* pipelines. Regarding *sleuth*, however, the results of the differential analysis itself are not bad and are even better than those of *Ballgown*, but when you go to also consider the unidentified genes or those detected despite not being expressed, the results collapse. In the case of the *kallisto* pipeline, in fact, it seems that pseudo-alignment leads to many more errors than traditional alignments. The *cummeRbund* pipeline proves to be the best in terms of specificity, with the lowest number of false positives, but *edgeR* and *DESeq2* overcome it in terms of sensitivity, with a lower number of false negatives. These last two pipelines present very similar results, as on the other hand we could expect due to the sharing of both the aligner and the quantification method. *edgeR* proves, however, slightly higher than *DESeq2*.

# **4. AUTOMATIC RNA-SEQ PIPELINES WITH INTERACTIVE REPORT (ARPIR)**

The automated pipeline developed by us consists of a series of bash, python and R scripts that allow a complete analysis from the quality control on FastQ files to the Pathway analysis.

The command can be launched via a graphical interface or via a command line. In the first case we are guided through a series of windows in the choice of files and input parameters and in particular:

- FastQ files of the samples. Since this is a differential analysis, at least two case and two control must be present, furthermore, if it is a paired-end analysis, files of read 1 and read 2 must be provided for each sample while, if it is a single-end analysis, only files of read 1 must be provided.
- Reference genome for *Bowtie* [31] with relative indexes, necessary for alignment with *TopHat*2.
- Reference genome for *HISAT2* with relative indexes, necessary for alignment with *HISAT2*.
- Reference genome for *STAR* with relative indexes, necessary for alignment with *STAR*.
- BED file, necessary for quality control on BAM files.
- PhiX genome with relative indexes, necessary for pre-processing.
- Ribosomal genome 1 (Human 5S ribosomal DNA) with relative indexes, necessary for pre-processing.
- Ribosomal genome 2 (Human ribosomal DNA) with relative indexes, necessary for pre-processing.
- GTF file, necessary for alignment with *TopHat2* and quantification with *featureCounts* and *Cufflinks*.
- Reference genome with relative indexes, necessary for quantification with *Cufflinks*.

To launch ARPIR from the command line, instead, we have created a script run.sh where you can enter all the desired variables. Once done, you can launch the pipeline with the following command:

bash run.sh

Once launched, ARPIR performs in sequence: quality control on FastQ files, pre-processing, alignment, quality control on BAM files (primary-analysis), quantification, differential analysis (secondary-analysis) and the tertiary-analysis.

## **4.1 FastQ quality**

The quality control on FastQ files is made with *FastQ-Screen* [32] and *FastQC* [33].

*FastQ-Screen* is useful for evaluating the amount of contaminating genome. The output of *FastQ-Screen* (Figure S3) shows the percentage of DNA of sample reads mapped on different genomes and in particular human, murine, PhiX and ribosomal genomes. To calculate this percentage, 100,000 random reads are selected from the sample and they are sequenced on the reference genomes, then the result is multiplied by the rest of the genome.

*FastQC* is a software that returns a series of output plots (Figure S2) useful for evaluating the quality of the sequence.


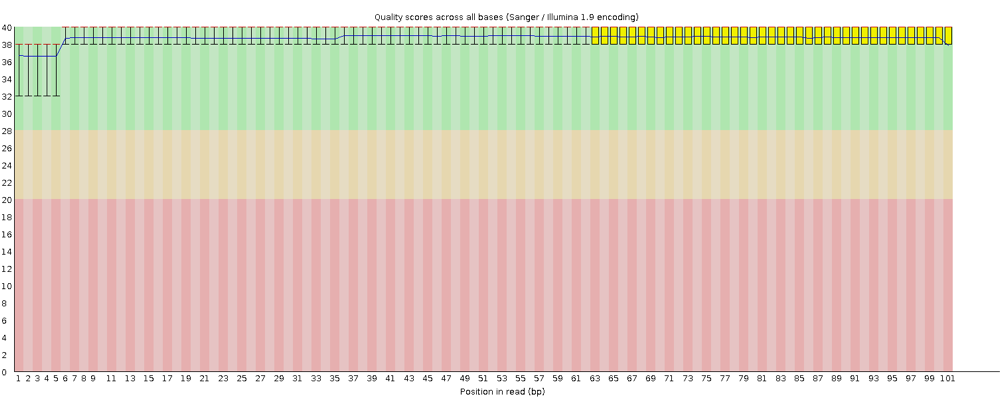


Figure S2. The plot is one of the outputs of FastQC and shows in the x axis the position of the base in the read, while in the y axis the value of the Quality Score. Values in the green band are considered good, in the yellow one they are acceptable and in the red one they indicate a low quality. For each base the red line indicates the value of the median, the yellow box represents the interquartile range (25-75%), the upper and lower bars represent the points at 10% and 90%, finally the blue line represents the average.


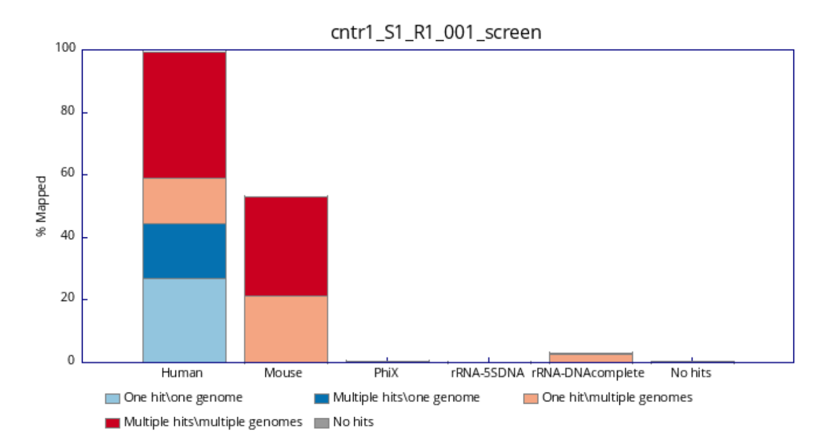


Figure S3. The plot is the output of FastQ-Screen and shows the percentage of read mapped on the human, murine, PhiX and ribosomal genome.

## **4.2 Pre-processing**

An initial quality control is made on the FastQ files with *FastQ-Screen* and *FastQC*, followed by a pre-processing to eliminate the reads of contaminating DNA, such as the PhiX genome and the ribosomal genome.

PhiX is a bacteriophage with a single-stranded DNA. Due to its small size (5,386 nucleotides) and its in-depth knowledge (it was the first DNA genome sequenced by Fred Sanger) the PhiX genome is used as a quality and calibration control for Illumina sequencing.

Despite the previous removal by specific kit of the contaminating RNA (above all rRNA), traces of RNA other than mRNA (and therefore not interesting for the purpose of the expression analysis) can remain however. For this reason, it is necessary to act also at the bioinformatics level to purify the data using a file with the RNA sequences to be removed.

An alignment of the reads with *bwa* on the PhiX and ribosomal genomes is performed with the following thresholds:

- -*k* [16] – Minimum seed length. Matches shorter than *k* will be missed. The alignment speed is usually insensitive to this value unless it significantly deviates 20.
- -*r* [1] – Trigger re-seeding for a MEM longer than minSeedLen. This is a key heuristic parameter for tuning the performance. Larger value yields fewer seeds, which leads to faster alignment speed but lower accuracy.
- -*M* – Mark shorter split hits as secondary (for Picard compatibility).
- -*T* [15] – Don't output alignment with score lower than *T*. This option only affects output.

Since in this case we are interested more in accuracy and less in speed, being still small genomes, we lowered the threshold of trigger re-seeding to 1 compared to the default value of 1.5 and for the same reason we lowered the minimum seed length to 16 compared to the default value of 19, to be sure not to lose any alignment. The threshold for the alignment score was also lowered to 15 compared to the default value of 30.

On the aligned reads a *samtools view* filter is then applied to distinguish only the best alignments, which are the DNA reads to be eliminated from the initial FastQ files. The parameters used are the following:

- -*F* [2308] – Do not output alignments with any bits set in INT present in the FLAG field. In this case, are deleted read unmapped, not primary alignment, supplementary alignment.
- -*q* [25] – Skip alignments with MAPQ smaller than *q*.
- -*f* [35] – Only output alignments with all bits set in INT present in the FLAG field. In this case, are kept read paired, read mapped in proper pair, mate reverse strand (this option is only present in case of paired-end reads).
- -*u* – Output uncompressed BAM. This option saves time spent on compression/decompression and is thus preferred when the output is piped to another *samtools* command.
- -*S* – Ignored for compatibility with previous *samtools* versions. Previously this option was required if input was in SAM format, but now the correct format is automatically detected by examining the first few characters of input.

What is obtained is a BAM file with sequences aligned on the PhiX and ribosomal genomes and that it is therefore necessary to remove from the original FastQ files. From the BAM files we then extract the headers of the sequences, used as input for the last step, which provides for their removal from the FastQ files. This is repeated both for the PhiX genome and for the two ribosomal genomes.

## **4.3 Alignment**

Alignment can occur with *TopHat2*, with *HISAT2* or with *STAR*.

*TopHat2* is an alignment software that provides an initial phase of alignment on the transcriptome, followed by the mapping of the remaining reads on the genome and the subsequent splicing alignment for the multi-exon sequences. The parameters used are the following:

- --*mate-inner-dist* [0] – This is the expected (mean) inner distance between mate pairs.
- --*mate-std-dev* [80] – The standard deviation for the distribution on inner distances between mate pairs.
- --*no-coverage-search* – Disables the coverage-based search for junctions.

The value of mate inner distance has been lowered to 0, which indicates filaments without space between them, attached or overlapped. This is because in the kit we used we have a distance between filaments smaller than the sequenced fragment, so it would not make sense to use an inner distance greater than 0.

The library-type option is used to specify the strand-specific information, that can be of three types: fr-unstrand, fr-firststrand, fr-secondstrand. The secondstrand means a read corresponds to a transcript, while the firststrand means a read corresponds to the reverse complemented counterpart of a transcript, while the unstrand both. The default option is fr-firststrand, which can be changed to fr-secondstrand among the initial options.

*HISAT2* is an alignment software that uses indexing schemes based on the Burrows-Wheeler transform and on the Ferragina-Manzini index (FM) using two types of alignment indexing: an FM index on the whole genome to anchor the various alignments and numerous local FM indexes for a rapid extension of these alignments. The parameters used are the following:

- --*dta* – Report alignments tailored for transcript assemblers including *StringTie*. With this option, *HISAT2* requires longer anchor lengths for de novo discovery of splice sites. This leads to fewer alignments with short-anchors, which helps transcript assemblers improve significantly in computation and memory usage.

The rna-strandness option is used to specify the strand-specific information, that can be of three types: unstrand, FR, RF. FR (corresponding to fr-secondstrand) means a read corresponds to a transcript, while RF (corresponding to fr-firststrand) means a read corresponds to the reverse complemented counterpart of a transcript, while the unstrand both. The default option is fr-firststrand (RF), which can be changed to fr-secondstrand (FR) among the initial options.

*STAR* (Spliced Transcripts Alignment to a Reference) software is based on a RNA-seq alignment algorithm that uses sequential maximum mappable seed search in uncompressed suffix arrays followed by seed clustering and stitching procedure.

## **4.4 BAM quality**

The quality of the BAM files is evaluated using *RSeQC* software [34], using a series of python scripts for creating different plots and in particular: *inner_distance*, which serves to measure the distribution of the internal distance between paired reads (Figure S4); *junction_annotation*, which determines which splice junctions are known, completely new or partially new based on the reference genome; *junction_saturation*, which determines whether the sequencing depth is sufficient for alternative splicing analysis; *bam_stat*, which makes statistics on read mapping; *read_distribution*, which calculates the fraction of reads mapped to the coding exon portion, the 5'-UTR region, the 3'-UTR region and the intron or intragenic regions.

Figure S4. One of the RSeQC outputs, the histogram of the distribution of the internal distance between paired reads.

## **4.5 Quantification**

Quantification can occur through *Cufflinks* or *featureCounts*.

*Cufflinks* is a software that, starting from the alignment output, proceeds first with the assembly of the transcripts, including all the different isoforms, then calculates the abundance and estimates the most likely by the value of likelihood.

*FeatureCounts* is a software that, starting from the alignment output, calculates the abundance of the transcripts using the simple method of reads count. The parameters used are the following:

- -*p* – If specified, fragments (or templates) will be counted instead of reads.

## **4.6 Differential Expression Analysis**

Differential analysis can occur through *cuffdiff* and *cummeRbund*, through *DESeq2* or through *edgeR*.

*Cuffdiff* is a software that, starting from the quantification of the transcripts operated by *Cufflinks*, calculates the levels of differential expression between different conditions by testing the log of the Fold Change observed against the null hypothesis of no change, which corresponds to a value of Fold Change equal to zero. The results obtained with *cuffdiff* were then processed with *cummeRbund*.

The R package *DESeq2* is based on a generalized linear model where the counts for each gene for each sample are modeled using a negative binomial distribution. The *p*-value for the differential expression between the different groups is calculated by a Wald test, which consists in dividing the value of log2-Fold Change estimated for each gene for the estimated error of the same; in this way a *Z* value is obtained which must be compared with the normal distribution to obtain the *p*-value. Finally, the *p*-value obtained is adjusted by the FDR method.

In the case of R package *edgeR*, before the differential analysis, the genes were filtered to keep those that have at least one count per million in at least two samples. After filtering, the data were normalized through the TMM method (Trimmed Mean of *M* values). The model used is a GLM (Generalized Linear Model), which represents an extension of the simplest linear model. Each gene is fitted through a negative binomial distribution. Differential expression analysis is performed by a likelihood ratio test. It consists in comparing the logarithm of the calculated likelihood for two different models, one of which is the one obtained under the null hypothesis. The value obtained is compared with the corresponding probability distribution and thus the *p*-value is obtained, which is then corrected by the FDR method.

Finally, a series of plots are generated. A PCA plot is created from the FPKM matrix calculated for the various samples with the help of the *ggfortify* package. From the results of the DEA instead a volcano plot is generated (using the *ggrepel* package) to show the Fold Changes and the p-values for all the genes (Figure S5). Finally, two heatmaps are created with the *gplots*, *RColorBrewer* and *genefilter* packages.


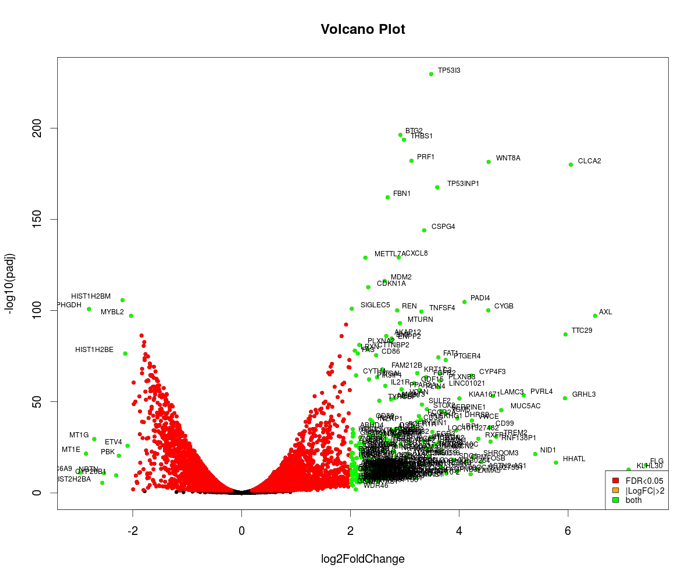


Figure S5. Representation of the log2-fold change and of the -log10 p-value. The genes in red are those that are significantly expressed, the orange ones the ones with Fold Change greater than 2 and the green ones the genes are significantly expressed and with Fold Change greater than 2.

## **4.7 Gene Ontology analysis**

The Gene Ontology database, developed by the GO consortium [35], allows to analyze genes on the basis of their molecular function, the biological process in which they intervene or the cellular localization of their products. One of the main uses of GO is to perform enrichment analysis on genes. For the analysis of GO, the R package *clusterProfiler* [36] is used, evaluating genes with an absolute value of Fold Change greater than 1.5 and an adjusted *p*-value of less than 0.05. The enrichment is evaluated with a *p*-value threshold of 0.05 and the adjustment method for multiple tests FDR.

We have resorted to the *org.Hs.eg.db* package [37] to map gene symbols to entrez and to the *rlist* package [38] to manage the results in lists. The *treemap* package [39] was used to create the plot, while for the dotplots and cnetplots we used the *DOSE* package [40] and a modified version of its *cnetplot* function (contained in *CNETPLOT_FUNCTION.R*), in addition to *igraph* [41] and *scales* [42] packages.

## **4.8 Pathway analysis**

Another method of gene clustering is to use the Pathway analysis in order to relate the genes obtained from previous analysis with the metabolic and signaling pathways present in public databases. One of the most used pathway databases is the Kyoto Encyclopedia of Genes and Genomes (KEGG) [43-45], which since its introduction in 1995 has proved a useful reference for the understanding of biological pathways. For the Pathway analysis, the R package *clusterProfiler* is used, evaluating genes with an absolute value of Fold Change greater than 1.5 and an adjusted *p*-value of less than 0.05. The enrichment is evaluated with a *p*-value threshold of 0.05 and the adjustment method for multiple tests FDR. The *pathview* package [46] was used to create the plots for the individual pathways enriched in KEGG.

## **4.9 Shiny report**

To observe the results of the analysis in an interactive form, it is possible to launch a *Shiny App* [47] with the following command:

R -e "shiny::runApp('/path/to/shiny/app/directory/',port=8080,host='localhost')" --args "/output_dir/project_name/pool_name"

It requires the path with the results folder as input argument, introduced via the --*args* option. The -*e* option is used to run the command to execute the script and then exit the R environment. “Port” and “host” indicate the address to which the App will be executed.

The *Shiny App* consists of two scripts, one in which the graphical interface is composed (*ui.R*) and another in which the content is processed (*server.R*).

The *ui.R* script composes the various tabs using the *navbarPage* function of *shiny* and the results are shown in the order that has already been shown in the main article. In the *server.R* script we used the *magick* package [48] to import and possibly modify the plots generated in *png* and *pdf* during the analysis. To show the tables in an interactive form, we instead resorted to the *DT* package [49]. The results shown are almost all the files generated during the analysis, with the sole exception of the interactive networks for *Pathway analysis* and *GO analysis* (Figure S6), which were created with the *visNetwork* package [50]. Finally, the downloadable report is generated through a *report.Rmd* file called within the *Shiny App* and the *render* function of *rmarkdown* [51].

**
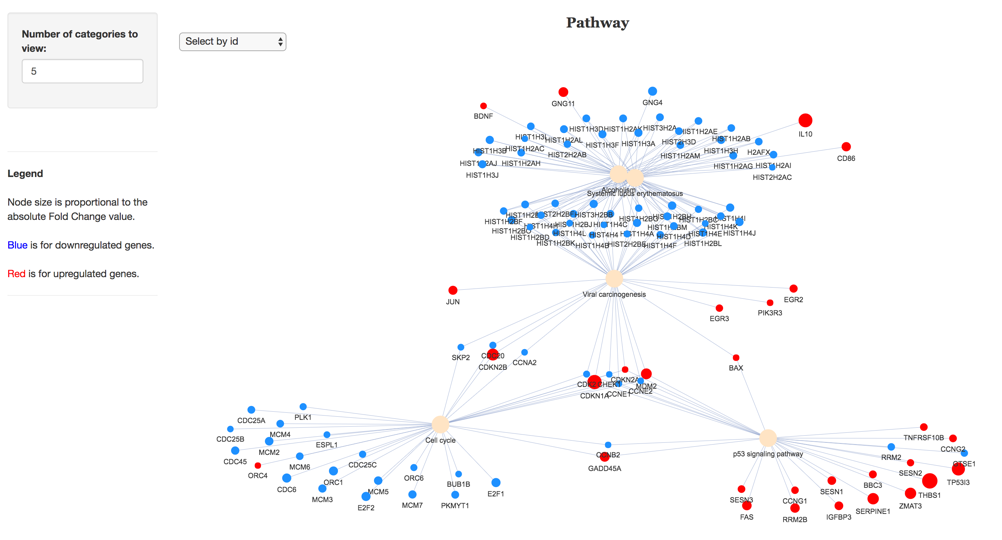
**

Figure S6. Interactive network generated for Pathway analysis. You can move and select the nodes as you wish and also change the number of enriched pathways shown.

# **REFERENCES**

1. **List of RNA-Seq bioinformatics tools** [<https://en.wikipedia.org/wiki/List_of_RNA-Seq_bioinformatics_tools>]

2. Goncalves A, Tikhonov A: **ArrayExpressHTS: ArrayExpress High Throughput Sequencing Processing Pipeline.** *R Package* 2019.

3. Torre D, Lachmann A, Ma'ayan A: **BioJupies: Automated Generation of Interactive Notebooks for RNA-Seq Data Analysis in the Cloud**. *Cell Syst* 2018, **7**(5):556-561 e553.

4. Kartashov AV, Barski A: **BioWardrobe: an integrated platform for analysis of epigenomics and transcriptomics data**. *Genome Biol* 2015, **16**:158.

5. Kallio MA, Tuimala JT, Hupponen T, Klemela P, Gentile M, Scheinin I, Koski M, Kaki J, Korpelainen EI: **Chipster: user-friendly analysis software for microarray and other high-throughput data**. *BMC Genomics* 2011, **12**:507.

6. Lopez-Fernandez H, Blanco-Miguez A, Fdez-Riverola F, Sanchez B, Lourenco A: **DEWE: A novel tool for executing differential expression RNA-Seq workflows in biomedical research**. *Comput Biol Med* 2019, **107**:197-205.

7. Cumbie JS, Kimbrel JA, Di Y, Schafer DW, Wilhelm LJ, Fox SE, Sullivan CM, Curzon AD, Carrington JC, Mockler TC *et al*: **GENE-counter: a computational pipeline for the analysis of RNA-Seq data for gene expression differences**. *PLoS One* 2011, **6**(10):e25279.

8. **GT-FAR** [<https://genomics.isi.edu/gtfar>]

9. Lohse M, Bolger AM, Nagel A, Fernie AR, Lunn JE, Stitt M, Usadel B: **RobiNA: a user-friendly, integrated software solution for RNA-Seq-based transcriptomics**. *Nucleic Acids Res* 2012, **40**(Web Server issue):W622-627.

10. Wang Y, Mehta G, Mayani R, Lu J, Souaiaia T, Chen Y, Clark A, Yoon HJ, Wan L, Evgrafov OV *et al*: **RseqFlow: workflows for RNA-Seq data analysis**. *Bioinformatics* 2011, **27**(18):2598-2600.

11. Wolfien M, Rimmbach C, Schmitz U, Jung JJ, Krebs S, Steinhoff G, David R, Wolkenhauer O: **TRAPLINE: a standardized and automated pipeline for RNA sequencing data analysis, evaluation and annotation**. *BMC Bioinformatics* 2016, **17**:21.

12. Chi HT, Vu HA, Iwasaki R, Nagamura F, Tojo A, Watanabe T, Sato Y: **Detection of exon 12 type A mutation of NPM1 gene in IMS-M2 cell line**. *Leuk Res* 2010, **34**(2):261-262.

13. Illumina: **TruSeqTM RNA and DNA Sample Preparation Kits v2**. *Data Sheet Illumina® Seq* 2011:1-4.

14. Illumina: **HiSeq® 2500 Sequencing System**. *Specif Sheet Illumina® Seq* 2015:1-4.

15. Kim D, Pertea G, Trapnell C, Pimentel H, Kelley R, Salzberg SL: **TopHat2: accurate alignment of transcriptomes in the presence of insertions, deletions and gene fusions**. *Genome Biol* 2013, **14**(4):R36.

16. Trapnell C, Williams BA, Pertea G, Mortazavi A, Kwan G, van Baren MJ, Salzberg SL, Wold BJ, Pachter L: **Transcript assembly and quantification by RNA-Seq reveals unannotated transcripts and isoform switching during cell differentiation**. *Nat Biotechnol* 2010, **28**(5):511-515.

17. **cummeRbund: Analysis, exploration, manipulation, and visualization of Cufflinks high-throughput sequencing data** [<https://bioconductor.org/packages/release/bioc/html/cummeRbund.html>]

18. Trapnell C, Hendrickson DG, Sauvageau M, Goff L, Rinn JL, Pachter L: **Differential analysis of gene regulation at transcript resolution with RNA-seq**. *Nat Biotechnol* 2013, **31**(1):46-53.

19. Kim D, Langmead B, Salzberg SL: **HISAT: a fast spliced aligner with low memory requirements**. *Nat Methods* 2015, **12**(4):357-360.

20. Pertea M, Pertea GM, Antonescu CM, Chang TC, Mendell JT, Salzberg SL: **StringTie enables improved reconstruction of a transcriptome from RNA-seq reads**. *Nat Biotechnol* 2015, **33**(3):290-295.

21. **ballgown: Flexible, isoform-level differential expression analysis** [<https://www.bioconductor.org/packages/release/bioc/html/ballgown.html>]

22. Li H: **A statistical framework for SNP calling, mutation discovery, association mapping and population genetical parameter estimation from sequencing data**. *Bioinformatics* 2011, **27**(21):2987-2993.

23. Liao Y, Smyth GK, Shi W: **featureCounts: an efficient general purpose program for assigning sequence reads to genomic features**. *Bioinformatics* 2014, **30**(7):923-930.

24. Love MI, Huber W, Anders S: **Moderated estimation of fold change and dispersion for RNA-seq data with DESeq2**. *Genome Biol* 2014, **15**(12):550.

25. Robinson MD, McCarthy DJ, Smyth GK: **edgeR: a Bioconductor package for differential expression analysis of digital gene expression data**. *Bioinformatics* 2010, **26**(1):139-140.

26. McCarthy DJ, Chen Y, Smyth GK: **Differential expression analysis of multifactor RNA-Seq experiments with respect to biological variation**. *Nucleic Acids Res* 2012, **40**(10):4288-4297.

27. Dobin A, Davis CA, Schlesinger F, Drenkow J, Zaleski C, Jha S, Batut P, Chaisson M, Gingeras TR: **STAR: ultrafast universal RNA-seq aligner**. *Bioinformatics* 2013, **29**(1):15-21.

28. Bray NL, Pimentel H, Melsted P, Pachter L: **Near-optimal probabilistic RNA-seq quantification**. *Nat Biotechnol* 2016, **34**(5):525-527.

29. **sleuth: Tools for investigating RNA-Seq** [<https://pachterlab.github.io/sleuth/>]

30. Frazee AC, Jaffe AE, Langmead B, Leek JT: **Polyester: simulating RNA-seq datasets with differential transcript expression**. *Bioinformatics* 2015, **31**(17):2778-2784.

31. Langmead B, Trapnell C, Pop M, Salzberg SL: **Ultrafast and memory-efficient alignment of short DNA sequences to the human genome**. *Genome Biol* 2009, **10**(3):R25.

32. **FastQ Screen** [<http://www.bioinformatics.babraham.ac.uk/projects/fastq_screen/>]

33. **FastQC: A quality control tool for high throughput sequence data.** [<http://www.bioinformatics.babraham.ac.uk/projects/fastqc/>]

34. Wang L, Wang S, Li W: **RSeQC: quality control of RNA-seq experiments**. *Bioinformatics* 2012, **28**(16):2184-2185.

35. Ashburner M, Ball CA, Blake JA, Botstein D, Butler H, Cherry JM, Davis AP, Dolinski K, Dwight SS, Eppig JT *et al*: **Gene ontology: tool for the unification of biology. The Gene Ontology Consortium**. *Nat Genet* 2000, **25**(1):25-29.

36. Yu G, Wang LG, Han Y, He QY: **clusterProfiler: an R package for comparing biological themes among gene clusters**. *OMICS* 2012, **16**(5):284-287.

37. **org.Hs.eg.db: Genome wide annotation for Human** [<https://bioconductor.org/packages/release/data/annotation/html/org.Hs.eg.db.html>]

38. **rlist: A Toolbox for Non-Tabular Data Manipulation** [<https://CRAN.R-project.org/package=rlist>]

39. **treemap: Treemap Visualization** [<https://CRAN.R-project.org/package=treemap>]

40. Yu G, Wang LG, Yan GR, He QY: **DOSE: an R/Bioconductor package for disease ontology semantic and enrichment analysis**. *Bioinformatics* 2015, **31**(4):608-609.

41. Csardi G, Nepusz T: **The igraph software package for complex network research**. *InterJournal* 2006, **Complex Systems**:1695.

42. **scales: Scale Functions for Visualization** [<https://CRAN.R-project.org/package=scales>]

43. Kanehisa M, Furumichi M, Tanabe M, Sato Y, Morishima K: **KEGG: new perspectives on genomes, pathways, diseases and drugs**. *Nucleic Acids Res* 2017, **45**(D1):D353-D361.

44. Kanehisa M, Goto S: **KEGG: kyoto encyclopedia of genes and genomes**. *Nucleic Acids Res* 2000, **28**(1):27-30.

45. Kanehisa M, Sato Y, Kawashima M, Furumichi M, Tanabe M: **KEGG as a reference resource for gene and protein annotation**. *Nucleic Acids Res* 2016, **44**(D1):D457-462.

46. Luo W, Brouwer C: **Pathview: an R/Bioconductor package for pathway-based data integration and visualization**. *Bioinformatics* 2013, **29**(14):1830-1831.

47. **shiny: Web Application Framework for R.** [<https://cran.r-project.org/web/packages/shiny/index.html>]

48. **magick: Advanced Graphics and Image-Processing in R.** [<https://CRAN.R-project.org/package=magick>]

49. **DT: A Wrapper of the JavaScript Library 'DataTables'** [<https://CRAN.R-project.org/package=DT>]

50. **visNetwork: Network Visualization using 'vis.js' Library.** [<https://CRAN.R-project.org/package=visNetwork>]

51. **rmarkdown: Dynamic Documents for R** [<https://CRAN.R-project.org/package=rmarkdown>]
