# Supplementary material for: ARPIR: automatic RNA-Seq pipelines with interactive report
Source: BMC Bioinformatics. 2020 Dec 21;21(Suppl 19):574. doi: 10.1186/s12859-020-03846-2 (PMC7751108; doi:10.1186/s12859-020-03846-2)
Supplement: Supplementary file 11 — Additional file 11. ARPIR User guide. [file 12859_2020_3846_MOESM11_ESM.docx]

**ARPIR: Automatic RNA-Seq Pipelines with Interactive Report**

Giulio Spinozzi^1*^, Valentina Tini^1^, Alessia Adorni^1^, Brunangelo Falini^1^ and Maria Paola Martelli^1*^

^1^Department of Medicine, Section of Hematology, University of Perugia, Perugia, Italy

*Email of Corresponding authors: [giulio.spinozzi@unipg.it](mailto:giulio.spinozzi@unipg.it), [maria.martelli@unipg.it](mailto:maria.martelli@unipg.it)

**Abstract**

**Background**

RNA-Seq is an increasing used methodology to study either coding and non-coding RNA expression. There are many software tools available for each phase of the RNA-Seq analysis and each of them uses different algorithms. Furthermore, the analysis consists of several steps regarding alignment (*primary-analysis*), quantification, differential analysis (*secondary-analysis*) and any *tertiary-analysis* and can therefore be time-consuming to deal with each step separately, in addition to requiring a computer knowledge. For this reason, the development of an automated pipeline that allows the entire analysis to be managed through a single initial command and that is easy to use even for those without computer skills can be useful. Faced with the vast availability of RNA-Seq analysis tools, it is first of all necessary to select a limited number of pipelines to include. For this purpose, we compared eight pipelines obtained by combining the most used tools and for each one we evaluated peak of RAM, time, sensitivity and specificity.

**Results**

The pipeline with shorter times, lower consumption of RAM and higher sensitivity is the one consisting in *HISAT2* for alignment, *featureCounts* for quantification and *edgeR* for differential analysis. Here, we developed ARPIR, an automated pipeline that recurs by default to the cited pipeline, but it also allows to choose, between different tools, those of the pipelines having the best performances.

**Conclusions**

ARPIR allows the analysis of RNA-Seq data from groups undergoing different treatment allowing multiple comparisons in a single launch and can be used either for paired-end or single-end analysis. All the required prerequisites can be installed via a configuration script and the analysis can be launched via a graphical interface or by a template script. In addition, ARPIR makes a final tertiary-analysis that includes a *Gene Ontology* and *Pathway analysis*. The results can be viewed in an interactive *Shiny App* and exported in a report (*pdf*, *word* or *html* formats). ARPIR is an efficient and easy-to-use tool for RNA-Seq analysis from quality control to *Pathway analysis* that allows you to choose between different pipelines.

**Keywords**

RNA-Seq

Bioinformatics

Pipelines

Genomics

DEA

Pathways

Gene Ontology

**Background**

RNA-Seq is a technology for the study of the transcriptome based on next-generation sequencing (NGS). Developed since the 2000s, it quickly became one of the methods of choice in the study of differential expression in various fields. One of these is the study of tumors and among them the leukemias, including acute myeloid leukemias (AML), where the RNA-Seq is used with increasing frequency either to characterize the disease or for diagnostic and risk assessment prognosis [1]. For this reason, it becomes important to use with efficiency and simplicity the tools that allow to operate standard analysis, from differential analysis to *Pathway* and *Gene Ontology analyses*. An automated pipeline would be useful for this purpose and would save time for analysis.

There are already many pipelines for the analysis of RNA-Seq data, but often they do not include final tertiary-analysis or quality control or there is no possibility to explore the results as a whole through an interactive report. Evaluating the 29 pipelines cited on Wikipedia [2] we noticed that none met all main requirements that could be of interest for an RNA-Seq analysis (see Additional File 1). For this reason, we decided to develop our own pipeline that was as complete as possible in dealing with each step.

Given the number of tools available, the first step was to understand which ones to introduce into the pipeline. In fact, having the possibility to choose between different pipelines can be useful, but at the same time having too many choices can be counterproductive, also because it requires a higher number of prerequisites to install and consequently also a greater storage space. We have therefore selected eight of the most used pipelines in the RNA-Seq analysis and we have chosen six based on the results of sensitivity and specificity obtained on simulation data, as well as for peaks of RAM reached and time taken. The aim is not to evaluate which are the best tools in the different phases of the analysis, as other papers have already dealt with these aspects [3-7] ​​and for this reason we have selected pipelines already published, but simply choose a small number of pipelines to include in the tool taking into consideration generic and computational aspects.

**Implementation**

Before using ARPIR, it is necessary to install a series of prerequisites and for this purpose a configuration script is provided which can be launched on any Debian-based system. In other cases, the script must be edited or it is possible to resort to manual installation.

ARPIR can be launched through a graphical interface, developed through the *zenity* [8] software in a bash script *GUI_ARPIR.sh*. Once the interface has been started, in the first window you are asked to enter: project name, which will be the name of the main folder; pool name, which represents a subfolder in which the results will be written; sample names, separated by comma, without spaces and ordered to have first the controls and then the treated (both at least in duplicate); sample types, in the same order of names and separated by comma, represent the belonging of the samples to one or the other group; log name, that is the name of the file in which the analysis log will be saved; comparisons of interest. In the second window it should be specified if it is a paired-end or single-end analysis. It is then requested to select, in order: the read 1 and any read 2 belonging to the various samples, the reference genome (and related indexes), the BED file, the genome PhiX (a control genome commonly used as a control for Illumina sequencing runs), the ribosomal genome 1 (5s rDNA) and 2 (rDNA), the GTF file, the reference genome, the directory where the scripts are located, the output directory, the directory in which to save the log, the library type, the pipeline desired for alignment, quantification and DEA (Differential Expression Analysis), if you want to make a final tertiary-analysis, and if so, the number of categories you want to display in the final plots (numbers above five tend to decrease readability), the number of threads to use. Finally, there is a summary table to check all inputs and parameters entered.

For launching from the command line the same information is required. The command in this case can be launched using a template (see Additional File 1).

**The Pipeline**

The main analysis is the Python script *ARPIR.py* (Figure 1), in which all the various scripts are recalled, with the exception of the *Shiny App*, which can be launched from the command line once the analysis is complete.

After a series of checks to verify the existence of the input files and the correctness of the parameters, the first script called is the alignment (*alignment.sh*, *alignment_se.sh* for single-end). In addition, an input.csv file is created in which the sample information will be saved, which are necessary for subsequent analysis. The alignment script acts on one sample at a time, in the order in which they are inserted.

Before the actual alignment, a quality control is performed with *FastQC* [9] on the supplied files and the report for the various samples is saved in the “Quality” folder. Then the analysis with *FastQ Screen* [10] follows to identify the genome contaminant and also in this case the results are saved in the “Quality” folder.

Then the pre-processing phase for PhiX and ribosomal genome removal begins. Through *bwa mem* [11] the FastQ files are aligned on the contaminant genome and the reads filtered with *samtools* [12, 13] to keep only the best alignments. The procedure is repeated both for the PhiX genome and for the ribosomal genome (for each sample).

Then the actual alignment phase follows, which can be performed with *TopHat2* [14], *HISAT2* [15] and STAR [16]. On the BAM files obtained, a quality control is performed using *RSeQC* [17], in particular by using *inner distance*, *junction annotation*, *junction saturation*, *bam statistics*, and *read distribution*. The results are saved in a “RSeQC” folder inside the folder with the BAM files.

In addition to the BAM files, bigwig and bedgraph files are also produced in the same folder; they are useful to generate tracks that can be loaded – for visual inspection and comparison analysis – on genome browsers (e.g. UCSC Genome Browser, IGV and similar).

In this phase, two summary tables are also generated, saved in the “report” folder, with information regarding both the parameters used and the reads removed from the samples during the pre-processing.

The next script that is invoked is the one for quantification and DEA, *quantification.py*. Quantification can occur through *featureCounts* [18] or *Cufflinks* and in both cases the data contained in the input.csv file are used to trace the metadata and path of the samples. In the case of *Cufflinks*, quantification takes place through a series of successive steps that involve the *cufflinks*, *cuffmerge* and *cuffquant* functions and to these follows the differential analysis phase with *cuffdiff* (as described in [19]).

The R scripts for the final DEA analysis are then called. In the case of *featureCounts*, *runedgeR.R* or *runDESeq2.R* can be called and in the case of *Cufflinks* *runcummeRbund.R*. In all three cases, first the FPKM matrix for the samples is calculated and used to create the PCA plot, saved in the “Quantification and DEA” folder both in *png* and in *pdf* format.

Then the actual differential analysis follows. For *edgeR* [20], we first have a selection of genes that are represented at least one counts per million (CPM) in at least two samples; the filtering is performed independently of which sample belongs to which group so that no bias is introduced. Then follows a normalization through the Trimmed Mean of M values (TMM) method, an estimate of the dispersions (in order common, trended and tagwise dispersions) and finally the differential analysis, with a first fit to the Generalized Linear Model (GLM) followed by a Likelihood Ratio Test (LRT). TMM is the recommended for most RNA-Seq data where the majority (more than half) of the genes are believed not differentially expressed between any pair of the samples [21]; for this reason it is the default method used for normalization in *edgeR*. For *DESeq2* [22] a predefined analysis is performed through the steps: estimation of size factors, estimation of dispersion, and Negative Binomial GLM fitting and Wald statistics. The results are then subjected to a logarithmic transformation. As already mentioned, in the *Cufflinks* pipeline the differential analysis has already been done, so *cummeRbund* is used only to import and make the results readable. Finally, in all three cases the results are written in tables in *csv* format, where we can find the name of the gene, the value of *log2-Fold Change*, the adjusted *p*-value and the values ​​of FPKM for the various samples. A series of plots are also created and saved in *png* and *pdf* format: a *volcano plot*, showing the *Fold Change* and the *p*-value for all the genes, a *heatmap* of the 100 genes with greater variance for all the samples, a *correlation heatmap* between the samples, to verify the similarity between replicates and between samples subjected to different treatment.

The last invoked script is *GO_pathway.R* for the tertiary-analysis. An enrichment analysis is performed in the *Gene Ontology* database [23] on genes with an adjusted *p*-value of less than 0.05 and an absolute value of *log2-Fold Change* greater than 1.5 using the *clusterProfiler* package [24]. The results are saved in two tables: one presents the terms of Gene Ontology enriched in order of *p*-value and the list of genes present in each group, with the relative information of *p*-value, *q*-value and count; the other presents a gene enriched by row, the GO term for which it is enriched and the value of *Fold Change*. Furthermore, three types of graphs are generated: a *treemap* with all the terms of GO enriched and in which the dimensions are proportional to the number of genes; three *dotplots* (one for each GO domain), in which the five (default) most enriched categories are shown; three *cnetplots*, in which the same categories and their genes are shown in the form of networks. Then the *Pathway analysis* follows, performed using an enrichment test (via the *clusterProfiler* package) in the KEGG database [25-27]. Even in this case the results are saved in a csv table and in a *dotplot* and in a *cnetplot*. Furthermore, images of the most enriched pathways are generated with the *Fold Changes* of genes through the *pathview* package [28].

All results are saved in specific folders with the tables in *csv* and the plots in *pdf* and *png*.

To make the pipeline automated and not require a massive user intervention in the choice of parameters, we have made ARPIR a rigid pipeline, in which it is not possible to add tools or methods other than those provided.

**ShinyApp and Reporting**

You can view them interactively via the *Shiny App* with the command:

R -e "shiny::runApp('/path/to/shiny/app/directory/',port=8080,host='localhost')" --args "/output_dir/project_name/pool_name"

The results are shown in a series of tabs divided according to the analysis step (see Additional File 1 for further details) and the whole can be downloaded in a *pdf*, *docx* or *html* report. In addition to the results, the report also includes the parameters used and a brief description of the various steps taken during the analysis.

The summary tab of the *Shiny App* contains two tables. The first table shows the parameters chosen for the analysis, while the second shows a summary for the various samples. In particular, for each sample is reported the type (control or treated), the number of raw reads, which are the reads in the initial FastQ file, the number of PhiX reads, which are the reads removed because they are part of PhiX contaminating genome, and the number of ribosomal reads, which are instead the reads removed because belonging to the ribosomal RNA.

The FastQ quality tab contains the plots obtained from the quality analysis carried out with *FastQ-Screen* and *FastQC* on the various samples. The first plot is the output of *FastQ-Screen* and shows the percentage of DNA of sample reads mapped on human, murine, PhiX and ribosomal genomes. The second plot is instead the output of *FastQC* and shows the quality of the reads of the sample calculated using the Phred Score contained in the FastQ files. The quality is showed through boxplots based on the position on the read. The values found in the green zone are considered of good quality. The drop-down menu in sidebar allows to browse through the various samples.

The quality of the BAM files was evaluated using *RSeQC* software. In particular, in the first table statistics related to the reads mapping are reported, in the second table reads fractions mapped on the coding exon part, on the 5'-UTR region, on the 3'-UTR region and on the intronic or intragenic regions are reported. The first image shows the distribution of the internal distance between paired reads; the second image shows number of splicing junctions per percent of total reads and splits junctions in all, known and novel; the third and the fourth images show percentage of novel splicing junctions and events.

For the differential expression analysis, a summary table with the results and FPKM values for each sample is given. Then a series of summary plots follows and in particular: a *PCA* to evaluate the differences between the samples; a *volcano plot*, which reports the values of Fold Change and *p*-value for all genes; a *heatmap* of the 100 genes with greater variance, in which the value of the *Z*-score is reported and therefore the distance from the mean for the various samples; a *heatmap* showing the distances between the samples, calculated in a distance matrix using the Euclidean distance method.

The outputs of the *Gene Ontology analysis* are two summary tables, the first one showing a GO term for each line while the second one a gene for each line. Then three interactive networks follow, one for each category of GO, which allow to view the enriched genes and their Fold Change. Finally, there are a *treemap*, where the size of each rectangle is proportional to the number of genes, and three *dotplots*, which report the five terms of GO that were more enriched for the genes.

For the *Pathway analysis* a summary table with enriched pathways is shown, followed by an interactive network similar to that of GO. Finally, there is a *dotplot* with the five most enriched pathways.

**Materials and Methods**

The *in vitro* data we analyzed came from RNA-Seq experiments performed on two different AML cell lines with *NPM1* mutation: OCI-AML3 [29] and IMS-M2 [30]. In both cases the treatment conditions were compared with the conditions without treatment. For each condition the experiment was done in triplicate. The kit used for the preparation of the sample was the TruSeq RNA [31], while the sequencer used for the sequencing was HiSeq 2500 by Illumina [32] in rapid run and with a flow cell. Sequencing occurred in paired-end and using two lanes for sample. The two lanes corresponding to the same sample have been merged into a single file before the alignment phase.

To validate the results of DEA, we used also *in silico* RNA-Seq data. We have resorted to the R package *polyester* [8] to generate a set of samples in paired-end belonging to two different groups and with three replicates per group (see Additional File 2). The FastQ files thus obtained were analyzed through the eight pipelines until the differential expression data were obtained (see Additional File 3, 4, 5, 6, 7, 8, 9, 10). To obtain sensitivity and specificity values that were representative of the entire pipeline, we also considered missing alignments and false alignments [33] (see Additional file 1 for more details).

Both the data obtained from the cell line samples and the simulated RNA-Seq data were submitted to the same analysis.

An initial quality analysis was performed on FastQ files using *FastQC* [9] software and a contaminant genome evaluation using *FastQ-Screen* [10]. We then removed the PhiX genome and the ribosomal genome by identifying sequences through alignment on samples with *bwa*.

The eight pipelines we have considered are those that use some of the most popular tools (Figure 2). In particular, for alignment we considered three different aligners: *TopHat2* [14], *HISAT2* [15], STAR [16] and *kallisto* [34]. The first three are based on a classical alignment performed on the genome through different algorithms, while the fourth one uses a pseudo-alignment on the transcriptome. As for quantification, we have selected four software: *Cufflinks* [35], *StringTie* [36], *featureCounts* [18] and *kallisto* [34] itself, which in addition to the alignment also performs quantification. The first two resort to a statistical approach to quantification, while the third resort to a simple read count. Finally, for the differential analysis we used five different R packages: *cummeRbund* [37], *Ballgown* [38], *DESeq2* [39], *edgeR* [20] and *sleuth* [40]. Each uses a different statistical approach, except *cummeRbund* which merely shows the results, while the true analysis is performed by *Cufflinks*.

**Results and discussion**

For the eight pipelines, we analyzed the time taken for the various processes and the peaks of memory used. The data in Table 1 show the values for the analysis in the pipelines using 4 threads per process. The data shown are related to the analysis on the 6 samples of the IMS-M2 cell line (see Additional File 1 for more details).

**Table 1.** Table of the times and the RAM memory peaks reached during the RNA-Seq analysis for the eight pipelines.

|  | **Alignment** | **Quantification** | **DEA** | **Time** | **RAM** |
| --- | --- | --- | --- | --- | --- |
| **1° pipeline** | TopHat2 | Cufflinks | cummeRbund | 35h | 3.3Gb |
| **2° pipeline** | Hisat2 | StringTie | Ballgown | 4h | 4.3Gb |
| **3° pipeline** | Hisat2 | featureCounts | DESeq2 | 4h | 4.3Gb |
| **4° pipeline** | Hisat2 | featureCounts | edgeR | 4h | 4.3Gb |
| **5° pipeline** | STAR | Cufflinks | cummeRbund | 5h | 30Gb |
| **6° pipeline** | STAR | featureCounts | DESeq2 | 4h | 30Gb |
| **7° pipeline** | STAR | featureCounts | edgeR | 4h | 30Gb |
| **8° pipeline** | Kallisto | Kallisto | Sleuth | 2h | 2.2Gb |

The longest pipeline was found to be TopHat2-Cufflinks-cummeRbund, while the shorter kallisto-sleuth. The RAM memory peaks are maintained in all cases except for STAR pipelines below 5Gb.

By first evaluating the pipelines in terms of time consumption it was found that the slowest pipeline is that of *TopHat2* and the fastest is that of *kallisto*. From the point of view of memory consumption, all pipelines are quite similar. Interestingly, however, note that all the peaks are kept below 5Gb, so the analysis in all cases can be carried forward even on a normal PC. However, this is not the case with STAR, which requires 30Gb of RAM to work at its best.

As well as comparing time and RAM memory performance, we then compared the pipelines through the results obtained. We calculated sensitivity and specificity using the simulated RNA-Seq data (see Additional File 1 for more details) (Figure 3).

In the light of these results, it emerges that, although *kallisto* is the method that requires shorter times, the bad results in terms of sensitivity and specificity make it unreliable compared to other pipelines. Pipelines that use *STAR* or *HISAT2* for alignment and *featureCounts* for quantification are the ones that give better results, as they present the best values of sensitivity and specificity. In terms of time they require highly less time than the *TopHat2* pipeline. However, STAR pipelines are the ones that require more consumption of RAM and for this reason we preferred to choose one of the other pipelines as the default option, as they can also be launched on a normal PC. *edgeR* and *DESeq2* have very similar results both for time and for RAM peak and for sensitivity and specificity, but *edgeR* proves slightly higher in the differential analysis.

We have thus chosen to include in the tool *TopHat2-Cufflinks-cummeRbund*, *STAR-Cufflinks-cummeRbund*, *STAR-featureCounts-DESeq2*, *STAR-featureCounts-edgeR*, *HISAT2-featureCounts-DESeq2* and *HISAT2-featureCounts-edgeR* pipelines and to make the latter the default option (Figure 4).

**A**utomatic **R**NA-Seq **P**ipelines with **I**nteractive **R**eport (ARPIR) carry out the entire RNA-Seq analysis and can be used by command line, but a graphical interface is also available that, through a series of successive windows, allows to choose between different methods of alignment, quantification and differential expression analysis. In addition to the standard analysis, ARPIR also performs a series of quality controls and a pre-processing, as well as an optional final tertiary-analysis. In particular, it makes quality control on FastQ files, pre-processing, alignment, quality control on BAM files, transcript quantification and differential expression analysis. Given the input files and the working directory, ARPIR is completely automated. First, quality control on FastQ files is performed with *FastQC* e *FastQ-Screen*. *FastQC* makes quality control and creates one report for sample. *FastQ-Screen* estimates approximately the percentage of reads that can be mapped on genomes other than human, like ribosomal genome, PhiX genome and mouse genome. This allows to evaluate the presence of contaminating genomes. Pre-processing follows quality control: the reads are aligned on PhiX genome and ribosomal genome to eliminate contaminations. Alignment can be performed with *TopHat2*, *HISAT2* or *STAR*; in the first case quantification is performed with *Cufflinks* and DEA with *cummeRbund*, in the second case quantification is performed with *featureCounts* and DEA with *DESeq2* or *edgeR*, in the third case it is possible to choose one of the previous methods for quantification and DEA. A second intermediate quality control is also performed on the aligned BAM files with some of the *RSeQC* scripts and in particular: *inner distance, junction annotation, junction saturation, bam stat, read distribution*.

It is possible to perform an optional tertiary-analysis on the results. It consists in *Gene Ontology* enrichment and *KEGG* *Pathway* enrichment analyses on the differentially expressed genes (with absolute log2-Fold Change value higher than 1.5 and adjusted *p*-value lower than 0.05). The tertiary-analysis part has been developed exclusively for the human genome at the moment, although the rest of the pipeline can also work for different genomes. One of the future goals is to expand the genomes available also for tertiary-analysis.

Finally, the results obtained and saved in the appropriate folders can be viewed in an interactive *Shiny App* [41], from which you can also download a report with all the results. The advantage of showing the results in this form is that, once the *Shiny App* is launched, it is intuitive and easy to use even for those who are not familiar with computer science. The *Shiny App* shows the results of the RNA-Seq analysis divided into a series of tabs for each phase: the summary tab contains two tables that show the initial setting parameters and details about pre-processing on the FastQ files; the FastQ quality tab contains the *FastQC* and *FastQ-Screen* outputs; the BAM quality tab contains the *RSeQC* outputs obtained from the quality analysis on the aligned files; the differential expression analysis tab contains a result table and a series of plots and in particular a PCA, a volcano plot, a heatmap of the 100 genes with greater variance, a heatmap showing the distances between the samples; the Tertiary-analysis tab is divided into two sub-tab, one for *GO analysis* and the other for *Pathway analysis*, both containing a result table and a series of dotplots and interactive network plots (see Additional File 1 for more details).

Although ARPIR is not ready for High Performance Computing architecture (HPC), there are no limits for sample management. The memory management that ARPIR uses is very optimized and multiple sequencing runs can be managed in parallel, regardless of the number of samples.

RNA-Seq runs with 10-15 samples can be analyzed even in a normal personal computer with 8-16 GB of RAM. For larger runs, the use of a workstation with 64GB of RAM is recommended.

Other aspects of interest in the use of a tool for RNA-Seq analysis are reproducibility and transparency, important elements especially in view of scientific publications, where often it is required to describe in detail each step of the analysis [42-44]. However, this is not considered in most tools. The analysis performed with ARPIR is entirely reproducible since the code is open access and the individual steps are executed by different scripts, so that it is also possible to reproduce them separately. Furthermore, to offer the greatest possible clarity regarding the steps addressed, the log produced during the analysis is saved in the selected folder. Finally, in the markdown report that can be downloaded from the *Shiny App*, all the parameters used (both optional and default) are reported with an explanation of their function, as well as a brief description of each phase with related outputs.

**Conclusions**

In order to identify a reference pipeline for RNA-Seq analysis, we evaluated some of the most used tools by combining them in eight different pipelines. We considered consumption in terms of time and RAM memory and we also evaluated the sensitivity and specificity of the different pipelines through RNA-Seq simulation data. What has emerged is that the *HISAT2-featureCounts-edgeR* pipeline is the best in terms of time, RAM consumption and sensitivity, but good performances are also in the *HISAT2-featureCounts-DESeq2*, in the *TopHat2* and in the *STAR* pipelines.

We have therefore developed a single tool, ARPIR, which contains the six pipelines and automatically performs the entire standard analysis, from quality control on FastQ files to *Pathway* and *Gene Ontology analyses*. In addition, multiple comparisons can be made between different groups within a single run. In order to make ARPIR easier to use even for those not familiar with computer science, we have also provided a configuration script for installing prerequisites and the possibility of being launched through a graphical interface, as well as from the command line using a template script. Furthermore, to make the results more easily accessible, we have developed an interactive *Shiny App*, from which it is also possible to download a summary report. These features make ARPIR a complete tool, easy to use and a reference point for our institute in the field of RNA-Seq analysis [45].

**Availability and requirements**

**Project name:** ARPIR

**Project home page:** <https://github.com/giuliospinozzi/arpir>

**Operating systems:** Unix (Linux, Mac)

**Programming language:** Python, bash, R

**Other requirements:** zenity 3.18.1.1, Python 2.7.12 (modules: os, argparse, sys, csv, pandas), R 3.4.3 (packages: cummeRbund, edgeR, DESeq2, ggfortify, ggrepel, genefilter, RColorBrewer, gplots, clusterProfiler, dplyr, org.Hs.eg.db, igraph, scales, treemap, pathview, shiny, DT, magick, rlist, visNetwork, shinyjs, knitr), pandoc, multiqc 1.7, FastQC 0.11.5, FastQ Screen 0.11.3, bwa 0.7.12-r1039, samtools 1.9, pigz, GD::Graph Perl module, TopHat 2.1.1, RSeQC 2.6.4, HISAT 2.1.0, featureCounts 1.5.3, Cufflinks 2.2.1, STAR 2.5.0a

**License:** GNU GPL

**Any restrictions to use by non-academics:** no restrictions

**List of abbreviations**

**AML:** Acute Myeloid Leukemia

**BAM:** Binary Alignment Map

**bwa:** Burrows-Wheeler Aligner

**CPM:** Counts Per Million

**DEA:** Differential Expression Analysis

**edgeR:** Empirical Analysis of Digital Gene Expression Data in R

**FastQC:** FastQ Quality Control

**FDR:** False Discovery Rate

**FM index:** Ferragina-Manzini index

**FPKM:** Fragments Per Kilobase Million

**GLM:** Generalized Linear Model

**GO:** Gene Ontology

**GTF:** Gene transfer format

**GUI:** Graphical User Interface

**HISAT2:** Hierarchical Indexing for Spliced Alignment of Transcripts

**HPC:** High Performance Computing

**KEGG:** Kyoto Encyclopedia of Genes and Genomes

**LRT:** Likelihood Ratio Test

**NGS:** Next-Generation Sequencing

**NPM1:** Nucleophosmin 1

**PC:** Personal Computer

**PCA:** Principal Component Analysis

**RAM:** Random Access Memory

**RNA:** RiboNucleic Acid

**RNA-Seq:** RNA-Sequencing

**RSeQC:** RNA-Seq Quality Control

**SAM:** Sequence Alignment Map

**STAR:** Spliced Transcripts Alignment to a Reference

**TMM:** Trimmed Mean of M values

**Declarations**

**About this supplement**

This article has been published as part of BMC Bioinformatics Volume 21 Supplement 19 2020: Proceedings from the Joint NETTAB/BBCC 2019 Conference. The full contents of the supplement are available at <https://bmcbioinformatics.biomedcentral.com/articles/supplements/volume-21-supplement-19>

**Acknowledgements**

Laura Mincarelli and Francesca Milano for technical assistance to set up the RNA-Seq experiments.

**Funding**

This work has been supported by:

- European Research Council (ERC) to Maria Paola Martelli (ContraNPM1AML) (Consolidator Grant 2016 n. 725725) funded all the research, materials and activities needed for the production and analysis of data. Giulio Spinozzi holds a postdoc position and Alessia Adorni was a PhD student at the Department of Medicine, University of Perugia, both supported by ERC-Co2016 grant n. 725725
- ELIXIR-IIB (ELIX4_spinozzi) to Giulio Spinozzi supported all bioinformatic activities, in particular the intensive analyses of all simulations and for testing the other tools.
- CINECA (HP10CZ1LLU) to Giulio Spinozzi supported all bioinformatic activities, in particular the intensive analyses of all simulations and for testing the other tools.

Publication costs are funded by European Research Council (ERC) to Maria Paola Martelli (ContraNPM1AML) (Consolidator Grant 2016 n. 725725).

**Availability of data and materials**

The datasets supporting the conclusions of this article are included within the article (and its additional files). The software is available in the GitHub repository, (<https://github.com/giuliospinozzi/arpir>).

**Authors’ contributions**

GS conceived of the study, participated in its design and coordination. GS and VT created the application (ARPIR) and wrote the manuscript. AA created the Illumina library and sequenced the AML cell lines. GS, MPM and BF coordinated all work. All authors read and approved the final manuscript.

**Ethics approval and consent to participate**

Not applicable.

**Consent for publication**

Not applicable.

**Competing interests**

The authors declare that they have no competing interests.

**References**

1. Falini B, Martelli MP: **Impact of genomics in the clinical management of patients with cytogenetically normal acute myeloid leukemia**. *Best Pract Res Clin Haematol* 2015, **28**(2-3):90-97.

2. **List of RNA-Seq bioinformatics tools** [<https://en.wikipedia.org/wiki/List_of_RNA-Seq_bioinformatics_tools>], 21 May 2019.

3. Huang HC, Niu Y, Qin LX: **Differential Expression Analysis for RNA-Seq: An Overview of Statistical Methods and Computational Software**. *Cancer Inform* 2015, **14**(Suppl 1):57-67.

4. Costa-Silva J, Domingues D, Lopes FM: **RNA-Seq differential expression analysis: An extended review and a software tool**. *PLoS One* 2017, **12**(12):e0190152.

5. Rapaport F, Khanin R, Liang Y, Pirun M, Krek A, Zumbo P, Mason CE, Socci ND, Betel D: **Comprehensive evaluation of differential gene expression analysis methods for RNA-seq data**. *Genome Biol* 2013, **14**(9):R95.

6. Baruzzo G, Hayer KE, Kim EJ, Di Camillo B, FitzGerald GA, Grant GR: **Simulation-based comprehensive benchmarking of RNA-seq aligners**. *Nat Methods* 2017, **14**(2):135-139.

7. Germain PL, Vitriolo A, Adamo A, Laise P, Das V, Testa G: **RNAontheBENCH: computational and empirical resources for benchmarking RNAseq quantification and differential expression methods**. *Nucleic Acids Res* 2016, **44**(11):5054-5067.

8. Frazee AC, Jaffe AE, Langmead B, Leek JT: **Polyester: simulating RNA-seq datasets with differential transcript expression**. *Bioinformatics* 2015, **31**(17):2778-2784.

9. **FastQC: A quality control tool for high throughput sequence data.** [<http://www.bioinformatics.babraham.ac.uk/projects/fastqc/>], 10 Aug. 2018.

10. **FastQ Screen** [<http://www.bioinformatics.babraham.ac.uk/projects/fastq_screen/>], 10 Aug. 2018.

11. Li H, Durbin R: **Fast and accurate short read alignment with Burrows-Wheeler transform**. *Bioinformatics* 2009, **25**(14):1754-1760.

12. Li H: **A statistical framework for SNP calling, mutation discovery, association mapping and population genetical parameter estimation from sequencing data**. *Bioinformatics* 2011, **27**(21):2987-2993.

13. Li H, Handsaker B, Wysoker A, Fennell T, Ruan J, Homer N, Marth G, Abecasis G, Durbin R, Genome Project Data Processing S: **The Sequence Alignment/Map format and SAMtools**. *Bioinformatics* 2009, **25**(16):2078-2079.

14. Kim D, Pertea G, Trapnell C, Pimentel H, Kelley R, Salzberg SL: **TopHat2: accurate alignment of transcriptomes in the presence of insertions, deletions and gene fusions**. *Genome Biology* 2013, **14**(4):R36.

15. Kim D, Langmead B, Salzberg SL: **HISAT: a fast spliced aligner with low memory requirements**. *Nat Methods* 2015, **12**(4):357-360.

16. Dobin A, Davis CA, Schlesinger F, Drenkow J, Zaleski C, Jha S, Batut P, Chaisson M, Gingeras TR: **STAR: ultrafast universal RNA-seq aligner**. *Bioinformatics* 2013, **29**(1):15-21.

17. Wang L, Wang S, Li W: **RSeQC: quality control of RNA-seq experiments**. *Bioinformatics* 2012, **28**(16):2184-2185.

18. Liao Y, Smyth GK, Shi W: **featureCounts: an efficient general purpose program for assigning sequence reads to genomic features**. *Bioinformatics* 2014, **30**(7):923-930.

19. Roberts A, Goff L, Pertea G, Kim D, Kelley DR, Pimentel H, Salzberg SL, Rinn JL, Pachter L, Trapnell C: **Differential gene and transcript expression analysis of RNA-seq experiments with TopHat and Cufflinks**. *Nature Protocols* 2012, **7**(3):562-578.

20. Robinson MD, McCarthy DJ, Smyth GK: **edgeR: a Bioconductor package for differential expression analysis of digital gene expression data**. *Bioinformatics* 2010, **26**(1):139-140.

21. Robinson MD, Oshlack A: **A scaling normalization method for differential expression analysis of RNA-seq data**. *Genome Biol* 2010, **11**(3):R25.

22. Love MI, Huber W, Anders S: **Moderated estimation of fold change and dispersion for RNA-seq data with DESeq2**. *Genome Biol* 2014, **15**(12):550.

23. Ashburner M, Ball CA, Blake JA, Botstein D, Butler H, Cherry JM, Davis AP, Dolinski K, Dwight SS, Eppig JT *et al*: **Gene ontology: tool for the unification of biology. The Gene Ontology Consortium**. *Nat Genet* 2000, **25**(1):25-29.

24. Yu G, Wang LG, Han Y, He QY: **clusterProfiler: an R package for comparing biological themes among gene clusters**. *OMICS* 2012, **16**(5):284-287.

25. Kanehisa M, Furumichi M, Tanabe M, Sato Y, Morishima K: **KEGG: new perspectives on genomes, pathways, diseases and drugs**. *Nucleic Acids Res* 2017, **45**(D1):D353-D361.

26. Kanehisa M, Goto S: **KEGG: kyoto encyclopedia of genes and genomes**. *Nucleic Acids Res* 2000, **28**(1):27-30.

27. Kanehisa M, Sato Y, Kawashima M, Furumichi M, Tanabe M: **KEGG as a reference resource for gene and protein annotation**. *Nucleic Acids Res* 2016, **44**(D1):D457-462.

28. Luo W, Brouwer C: **Pathview: an R/Bioconductor package for pathway-based data integration and visualization**. *Bioinformatics* 2013, **29**(14):1830-1831.

29. Quentmeier H, Martelli MP, Dirks WG, Bolli N, Liso A, Macleod RA, Nicoletti I, Mannucci R, Pucciarini A, Bigerna B *et al*: **Cell line OCI/AML3 bears exon-12 NPM gene mutation-A and cytoplasmic expression of nucleophosmin**. *Leukemia* 2005, **19**(10):1760-1767.

30. Chi HT, Vu HA, Iwasaki R, Nagamura F, Tojo A, Watanabe T, Sato Y: **Detection of exon 12 type A mutation of NPM1 gene in IMS-M2 cell line**. *Leuk Res* 2010, **34**(2):261-262.

31. Illumina: **TruSeqTM RNA and DNA Sample Preparation Kits v2. Data Sheet Illumina® Seq., 1–4**. In*.*; 2011.

32. Illumina: **HiSeq® 2500 Sequencing System. Specif. Sheet Illumina® Seq., 1–4**. In*.*; 2015.

33. Spinozzi G, Calabria A, Brasca S, Beretta S, Merelli I, Milanesi L, Montini E: **VISPA2: a scalable pipeline for high-throughput identification and annotation of vector integration sites**. *BMC bioinformatics* 2017, **18**(1):520.

34. Bray NL, Pimentel H, Melsted P, Pachter L: **Near-optimal probabilistic RNA-seq quantification**. *Nature Biotechnology* 2016, **34**(5):525-527.

35. Trapnell C, Williams BA, Pertea G, Mortazavi A, Kwan G, van Baren MJ, Salzberg SL, Wold BJ, Pachter L: **Transcript assembly and quantification by RNA-Seq reveals unannotated transcripts and isoform switching during cell differentiation**. *Nature Biotechnology* 2010, **28**(5):511-U174.

36. Pertea M, Pertea GM, Antonescu CM, Chang TC, Mendell JT, Salzberg SL: **StringTie enables improved reconstruction of a transcriptome from RNA-seq reads**. *Nat Biotechnol* 2015, **33**(3):290-295.

37. **cummeRbund: Analysis, exploration, manipulation, and visualization of Cufflinks high-throughput sequencing data. R Packag.** [<https://bioconductor.org/packages/release/bioc/html/cummeRbund.html>], 10 Dec. 2017.

38. Frazee AC, Pertea G, Jaffe AE, Langmead B, Salzberg SL, Leek JT: **Ballgown bridges the gap between transcriptome assembly and expression analysis**. *Nat Biotechnol* 2015, **33**(3):243-246.

39. Love MI, Huber W, Anders S: **Moderated estimation of fold change and dispersion for RNA-seq data with DESeq2**. *Genome Biology* 2014, **15**(12).

40. **sleuth: Tools for investigating RNA-Seq. R Packag. version 0.29.0** [<https://github.com/pachterlab/sleuth>], 10 Dec. 2017.

41. **shiny: Web Application Framework for R. R package version 1.1.0.** [<https://cran.r-project.org/web/packages/shiny/index.html>], 10 Dec. 2017.

42. Ince DC, Hatton L, Graham-Cumming J: **The case for open computer programs**. *Nature* 2012, **482**(7386):485-488.

43. Peng RD: **Reproducible research in computational science**. *Science* 2011, **334**(6060):1226-1227.

44. Gentleman R: **Reproducible research: a bioinformatics case study**. *Stat Appl Genet Mol Biol* 2005, **4**:Article2.

45. Sportoletti P, Celani L, Varasano E, Rossi R, Sorcini D, Rompietti C, Strozzini F, Del Papa B, Guarente V, Spinozzi G *et al*: **GATA1 epigenetic deregulation contributes to the development of AML with NPM1 and FLT3-ITD cooperating mutations**. *Leukemia* 2019.

**Figures, tables and additional files**

**Figure legends**

**Figure 1.** Workflow of ARPIR pipeline. The ARPIR pipeline, starting from the input files and parameters, performs an RNA-Seq analysis. First of all the primary-analysis, a quality control on the FastQ files occurs, followed by a pre-processing and alignment, which can be done through TopHat2, HISAT2 or STAR, finally there is a new quality control on the BAM files. The secondary-analysis is the quantification and differential analysis, which can follow the featureCounts-edgeR, featureCounts-DESeq2 or Cufflinks-cummeRbund pipelines. Then an optional tertiary-analysis follows, composed of a GO analysis and a Pathway analysis. The results obtained can then be viewed in a Shiny App and possibly downloaded to a report.

**Figure 2.** Plot of the eight tested pipelines. We tested different pipelines with the most used tools. We considered four aligners, four software for quantification and five R packages for differential analysis.

**Figure 3.** Sensitivity and specificity values for the eight pipelines. Sleuth and Ballgown give the worst results, while DESeq2 and edgeR are distinguished by the low number of false negatives (sensitivity) and cummeRbund due to the low number of false positives (specificity).

**Figure 4.** Workflow of HISAT2-featureCounts-edgeR pipeline. The alignment, launched in bash, requires FastQ files and the reference genome and leads to BAM files, which in turn become inputs for featureCounts that generates a count matrix. Finally, the differential analysis with edgeR generates a differential expression matrix.

**List of additional data files**

Additional file 1: **Supplementary Material.** Detailed description of the steps addressed during the analysis. (DOCX 7.1 Mb)

Additional file 2: **Table S1.** List of genes differentially expressed and relative Fold Changes for the two groups in the original data. (CSV 11 kb)

Additional file 3: **Table S2.** List of genes differentially expressed and relative Fold Changes identify by *HISAT2-StringTie-Ballgown* pipeline. (CSV 28 kb)

Additional file 4: **Table S3.** List of genes differentially expressed and relative Fold Changes identify by *TopHat2-Cufflinks-cummeRbund* pipeline. (CSV 15 kb)

Additional file 5: **Table S4.** List of genes differentially expressed and relative Fold Changes identify by *HISAT2-featureCounts-DESeq2* pipeline. (CSV 27 kb)

Additional file 6: **Table S5.** List of genes differentially expressed and relative Fold Changes identify by *HISAT2-featureCounts-edgeR* pipeline. (CSV 27 kb)

Additional file 7: **Table S6.** List of genes differentially expressed and relative Fold Changes identify by *kallisto-sleuth* pipeline. (CSV 28 kb)

Additional file 8: **Table S7.** List of genes differentially expressed and relative Fold Changes identify by *STAR-featureCounts-edgeR* pipeline. (CSV 30 kb)

Additional file 9: **Table S8.** List of genes differentially expressed and relative Fold Changes identify by *STAR-featureCounts-DESeq2* pipeline. (CSV 30 kb)

Additional file 10: **Table S9.** List of genes differentially expressed and relative Fold Changes identify by *STAR-Cufflinks-cummeRbund* pipeline. (CSV 21 kb)

Additional file 11: **Supplementary Material.** ARPIR User guide. (DOCX 3 Mb)
